# Mapping the Landscape, Knowledge Gaps, and Areas for Innovation in Brain Health and Dementia Research in Canada: Protocol for a Scoping Review of Reviews

**DOI:** 10.2196/79020

**Published:** 2026-02-13

**Authors:** Juanita-Dawne Bacsu, Kiana Mero, Megan E O'Connell, Megan Funk, Alixe Ménard, Myrna Norman, Sheila Blackstock, Jim Mann, Wendy Hulko, Melba Sheila D’Souza, Sarah Fraser

**Affiliations:** 1 School of Nursing, Thompson Rivers University, 805 TRU Way, Kamloops, BC, V2C 0C8, Canada; 2 Department of Psychology, Thompson Rivers University, Kamloops, BC, Canada; 3 Department of Psychology & Health Studies, University of Saskatchewan, Saskatoon, SK, Canada; 4 Interdisciplinary School of Health Sciences, Faculty of Health Sciences, University of Ottawa, Ottawa, ON, Canada; 5 National dementia advocate and person living with dementia, Maple Ridge, BC, Canada; 6 National dementia advocate and person living with dementia, Surrey, BC, Canada; 7 School of Social Work and Human Service, Thompson Rivers University, Kamloops, BC, Canada

**Keywords:** dementia, Alzheimer disease, prevention, treatment, quality of life, Canada

## Abstract

**Background:**

Dementia is one of Canada’s most pressing public health challenges, with rates expected to surge in response to the country’s aging population. Given the rapidly growing issue of dementia, understanding national research efforts is critical to prioritizing and advancing strategic directions in brain health and dementia research. Recently, the Canadian Institutes of Health Research awarded a 1-year funding grant from the Brain Health and Cognitive Impairment in Aging Research Initiative to map the scope of brain health and dementia research in Canada.

**Objective:**

This scoping review of reviews protocol aims to address this call by outlining the methodology that will be used for mapping the research landscape, documenting the knowledge gaps, and identifying areas of innovation to advance brain health and dementia research in Canada.

**Methods:**

Given the large volume of literature, a scoping review of Canadian-led reviews was selected as the most appropriate method because it would allow for a robust synthesis of nationally relevant research while mapping knowledge gaps and innovation. Our scoping review of reviews will follow the framework by Arksey and O’Malley along with the PRISMA-ScR (Preferred Reporting Items for Systematic Reviews and Meta-Analyses extension for Scoping Reviews) guidelines. The search will focus on peer-reviewed literature reviews published between January 1, 2020, and January 1, 2025, to capture the current state of knowledge since the national dementia strategy’s publication in 2019. This search will be conducted using 5 electronic databases: CINAHL, PubMed, PsycInfo, Scopus, and Web of Science. Our data extraction table will include the following categories: author, province, and year; aim; review timeline; method; theme; knowledge gaps; innovations; and findings. The data will be analyzed using a combination of deductive and inductive thematic analysis.

**Results:**

This protocol was registered on June 5, 2025, with the Open Science Framework. This study was funded by the Canadian Institutes of Health Research from November 2024 to November 2025. The anticipated timeline for the publication of the full scoping review of reviews is May 2026. The findings from this review will be shared through targeted knowledge mobilization activities such as presentations at national funding agency meetings, academic conferences, and community workshops.

**Conclusions:**

Our scoping review of reviews will provide a robust synthesis of the brain health and dementia research landscape, helping document critical knowledge gaps and identify areas for innovation. The results of this research will provide critical data to help inform strategic funding initiatives and future research directions. The findings from our scoping review will have implications for research funders, policymakers, community organizations, and researchers that are working to accelerate brain health and dementia research across Canada.

## Introduction

Dementia is one of Canada’s most pressing public health challenges, with rates expected to surge in response to the country’s aging population. It is estimated that 771,939 Canadians live with dementia [1], and approximately 487,000 of them have received a diagnosis [2]. Advancing age is the greatest risk factor for dementia [3], with risk increasing to approximately 25% in adults aged 85 years and older (1 in 4 people) [4]. In 2023, the number of people aged 65 years or older was approximately 7.6 million, nearly one-fifth of Canada’s entire population [5]. In Canada, over 414 new cases of dementia are diagnosed every day [1]. By 2030, it is estimated that almost 1 million people will live with Alzheimer disease or another form of dementia in this country [6].

The financial cost of dementia in Canada is substantial. In 2020, Canada’s total direct and indirect costs of dementia were an estimated CAD $40.1 billion (US $29.3 billion) per year, with an average cost of CAD $67,200 (US $49,034) per person living with dementia [7]. Moreover, friends and family contribute over 580 million hours of care each year to people living with dementia [1]. Dementia care requires a comprehensive approach, including access to a timely dementia diagnosis, information and resources on treatment options, and support services for people living with dementia and their care partners [8].

Dementia is a leading cause of disability among older adults in Canada and worldwide [9]. By 2050, it is projected that over 1.7 million Canadians will live with dementia [1]. Accordingly, *A Dementia Strategy for Canada: Together We Aspire* emphasizes the importance of dementia research, highlighting the need to make advancements in 3 theme areas: dementia prevention, advancing therapies, and improving quality of life for people with dementia and their care partners [10].

There is extensive research on brain health and dementia in Canada. An initial PubMed search covering the past 5 years (2020‐2025) using the terms “cognitive impairment,” “cognitive decline,” “dementia,” “dementias,” “Alzheimer’s Disease,” “Alzheimer’s,” “mild cognitive impairment,” “brain health,” and “Canadian [Affiliation] OR Canada [Affiliation]” yielded over 10,000 articles. Although numerous Canadian studies exist, there is a paucity of knowledge synthesizing of the brain health and dementia research landscape. Recently, the Canadian Institutes of Health Research awarded a 1-year funding grant from the Brain Health and Cognitive Impairment in Aging Research Initiative to map the scope of Canadian research on brain health and dementia [11].

This scoping review of reviews aims to address this call by mapping the research landscape, documenting knowledge gaps, and identifying areas of innovation to advance brain health and dementia research in Canada. Given the large volume of literature and the 1-year time frame of our funded study, a scoping review of Canadian-led reviews was selected as the most appropriate method because it will allow for a robust synthesis of nationally relevant research while mapping gaps and innovation. To provide a targeted and manageable approach, we will structure our review around Canada’s national dementia strategy’s 3 theme areas (eg, prevention, treatment, and quality of life) [10] while also ensuring that data are collected to identify research gaps and areas for innovation to inform strategic funding initiatives and future research priorities. The findings from our scoping review will have implications for research funders, policymakers, community organizations, and researchers that are working to elevate brain health and dementia research in Canada.

## Methods

### Scoping Review Framework

Canadian-led reviews were selected for this scoping review to maximize the national relevance of the findings and their applicability to mapping brain health and dementia research within the Canadian context. Scholars indicate that scoping reviews are highly useful for mapping large bodies of literature and identifying knowledge gaps to determine the breadth of the field [12,13]. Peters et al [13] note that scoping reviews are increasingly being used to inform decision-making and future research directions. This scoping review of reviews protocol was registered with the Open Science Framework (a5bsx) on June 5, 2025.

This scoping review of reviews will adhere to the framework by Arksey and O’Malley [14], comprising six steps: (1) establishing the research question, (2) exploration of relevant studies, (3) selection of studies, (4) extracting and charting the data, (5) synthesizing and reporting key findings, and (6) expert consultation. We will share our findings following the PRISMA-ScR (Preferred Reporting Items for Systematic Reviews and Meta-Analyses extension for Scoping Reviews) checklist [12].

### Step 1: Establishing the Research Question

Our scoping review will be structured around the dementia strategy of Canada’s 3 thematic areas: dementia prevention, advancing treatment and finding a cure, and enhancing the quality of life of people with dementia and their care partners [10]. This scoping review of reviews protocol will consist of primary and secondary research questions.

#### Primary Research Question

The primary research question is as follows: drawing on the national dementia strategy’s 3 research areas (dementia prevention, treatment, and quality of life [10]), what are the research landscape, knowledge gaps, and areas of innovation within brain health and dementia research in Canada?

#### Secondary Research Questions

The secondary research questions are as follows:

- In the area of dementia prevention, what are the existing knowledge landscape, research gaps, and areas for innovation in brain health and dementia research in Canada?
- In the area of advancing treatments and finding a cure, what are the existing knowledge landscape, research gaps, and areas for innovation in brain health and dementia research in Canada?
- In the area of enhancing quality of life, what are the existing knowledge landscape, research gaps, and areas for innovation in brain health and dementia research in Canada?

### Step 2: Exploration of Relevant Studies

#### Overview

Our search will review 5 electronic databases: CINAHL, PubMed, PsycInfo, Scopus, and Web of Science. In conducting scoping reviews, Peters et al [13] recommend the population, concept, and context (PCC) framework to help support the search strategy by providing meaningful inclusion criteria. Accordingly, our search will be guided by a list of keywords that are documented in our PCC table (Table 1). Moreover, the search string for the PubMed database will be as follows: ((“Literature review*” OR “Systematic Review*” OR “Scoping Review*” OR “Narrative Review*” OR “Review” OR “Rapid Review*” OR “Umbrella Review” OR “Meta-Analysis*” OR “Meta synthesis”) AND (“Cognitive Impairment” OR “Cognitive Decline” OR “Dementia*” OR “Dementias” OR “Alzheimer’s Disease*” OR “Mild Cognitive Impairment*” OR “Alzheimer’s” OR “Brain Health*“) AND (Canada[Affiliation] OR Canadian[Affiliation] OR Canadians[Affiliation] OR “in Canada”[Affiliation])).

The search time frame will focus on peer-reviewed literature reviews published between January 1, 2020, and January 1, 2025. This time frame was selected to focus specifically on the reviews published since the national dementia strategy of Canada was launched [10]. Additionally, we connected with a highly skilled librarian to review and assess our proposed databases and search strategy.

**Table 1.** Population, concept, and context criteria.

Element	Description	Keywords
Population	Adults with cognitive impairment or dementia	“Dementia,” “Dementias,” “Alzheimer’s Disease,” “Alzheimer’s,” “Cognitive impairment,” “Mild Cognitive Impairment,” “Brain Health,” “Cognitive Decline”
Concept	Literature review types	“Literature review,” “Systematic Review,” “Scoping Review,” “Narrative Review,” “Rapid Review,” “Umbrella Review,” “Meta-Analysis,” “Meta Synthesis”
Context	Canadian context for national relevance	“Canada [Author Affiliation],” “Canadian [Author Affiliation],” “Canadians [Author Affiliation],” “in Canada [Author Affiliation]”

#### Inclusion and Exclusion Criteria

Our inclusion criteria will consist of five components: (1) peer-reviewed literature reviews (systematic reviews, scoping reviews, narrative reviews, rapid reviews, umbrella reviews, meta-syntheses, or meta-analyses) that outline the search strategy; (2) a publication date between January 1, 2020, and January 1, 2025; (3) being written in the English or French languages; (4) first author with a Canadian affiliation to ensure national relevance; and (5) a focus on brain health and/or dementia research. The exclusion criteria will be gray literature and empirical research rather than reviews.

It is important to note that we recognize that umbrella reviews may overlap with other review types. To address this issue, umbrella reviews will only be included if they provide a clearly documented search strategy, which will allow us to identify and assess any overlap with other included reviews. Using this strategy will help us support methodological transparency while maintaining consistency across the different types of reviews.

### Step 3: Selection of Studies

The titles and abstracts of the extracted reviews will be independently screened by 2 reviewers against the inclusion criteria. The same 2 reviewers will independently conduct the full-text screening of the reviews. Covidence (Veritas Health Innovation) will be used to manage all the literature reviews obtained in our search [15]. To document the review of the literature, we will follow the PRISMA-ScR guidelines [12] and create a flowchart to show the data filtering process. Any discrepancies regarding a review article’s inclusion will be resolved through discussion among the 2 reviewers to achieve consensus. However, any disagreements in which consensus cannot be reached will involve a third reviewer to facilitate discussion and reach consensus through communication among the 3 researchers.

### Step 4: Extracting and Charting the Data

Covidence will be used to create a data extraction table that will aid in systematically mapping the data from the literature reviews. The data extraction table will include the following categories: author, province, and year; aim; timeline searched; method; theme (prevention, treatment, or quality of life); knowledge gaps; innovations; and findings (including conclusions). Two reviewers will conduct a pilot test of the data extraction table to ensure the clarity of the data extraction process.

### Step 5: Synthesizing and Reporting Key Findings

#### Overview

The data will be analyzed using a combination of deductive and inductive thematic analysis. Specifically, a deductive approach will be used in which each review will be coded according to the 3 pre-established themes in Canada’s national dementia strategy: dementia prevention, advancing treatments and finding a cure, and enhancing quality of life [10]. Inductive thematic analysis will be performed to identify the underlying themes that exist within the data. For example, this process will be guided by the inductive thematic analysis framework by Braun and Clarke [16] and will include (1) familiarization and immersion with the data (eg, reading and rereading the information in the data extraction table), (2) creating initial codes (eg, developing clear and descriptive code names to capture segments of informative text), (3) establishing themes (eg, creating themes through the use of theme piles by organizing the codes into meaningful groups), (4) reviewing the themes (eg, ensuring that the themes are not redundant, are clear, and capture all the existing data), and (5) naming and defining the themes (eg, ensuring clarity and uniqueness in the theme names and definitions to avoid repetition among the themes). Guided by the framework by Braun and Clarke [16], the full research team will work to develop and refine the themes by assessing whether each theme is clearly understandable or too complex, determining whether more themes are needed, assessing whether the data adequately support each theme, and ensuring that no themes have been missed. The findings from the scoping review of reviews will be reported by adhering to and following the PRISMA-ScR guidelines [12].

#### Trustworthiness

Guided by the criteria of trustworthiness by Lincoln and Guba [17], this study will include measures of dependability, credibility, and transparency. Dependability will be established by providing a comprehensive audit trail, such as our PCC table with our search terms and identifying clear inclusion criteria for other researchers looking to reproduce our scoping review process. Credibility will be achieved by using a transparent process for selecting studies and documenting each stage of the review using a PRISMA (Preferred Reporting Items for Systematic Reviews and Meta-Analyses) flowchart. Confirmability will be supported by having the same 2 researchers independently complete the title and abstract screening and the full-text review. As previously mentioned, a third reviewer will be brought in to handle any discrepancies during the data screening to further reduce issues related to researcher bias. Confirmability will also be supported by engaging in researcher triangulation and having the full research team involved in interpreting the data during the thematic analysis to minimize the potential for researcher bias.

### Step 6: Expert Consultation

Our research team includes 2 people living with dementia who are national dementia advocates. They are involved in numerous research projects across Canada and have presented in podcasts, webinars, conferences, and government meetings. In this project, these research partners will be involved in all stages of our research, from refining the scoping review question to collaborating in knowledge mobilization strategies. For example, our research partners with lived experience will be involved in the development and refinement of our themes during the thematic analysis process. Additionally, they will attend our quarterly full-team meetings. Honoraria will be provided to them for their invaluable knowledge, time, and insights shared on the project.

Historically, people with lived experience have not often been included in research teams and have had little involvement in guiding research questions [18]. However, the inclusion of people living with dementia is essential to our work as it contributes to a more robust and critical lens to evaluate the landscape of brain health and dementia research. Brett et al [19] note that incorporating lived experience in reviews can be used to collectively identify knowledge gaps, develop research questions, assess the practical implications of the topic, and recognize relevant areas for future research. Furthermore, our research team consists of multidisciplinary professionals from across Canada with expertise in population health, nursing, social work, sociology, epidemiology, psychology, and family medicine.

## Results

This protocol was registered on June 5, 2025, with the Open Science Framework. This study was funded by the Canadian Institutes of Health Research from November 2024 to November 2025. The scoping review’s data collection and analysis will be conducted from August 2025 to September 2025. The anticipated timeline for the publication of the full scoping review of reviews is spring May 2026. Our knowledge mobilization strategy will be developed to connect with multiple stakeholders, including funders, policymakers, researchers, and community organizations involved in supporting brain health and dementia research. We will tailor our dissemination approaches to target and align with each stakeholder group. For example, we will share a presentation with the members of the Dementia Research and Innovation Funders Alliance, as well as with policymakers during their annual meeting in Ottawa, Ontario, to inform strategic funding directions and priorities [20]. Additionally, findings will be shared at academic conferences and in journal articles to target researchers. Newsletter articles and community workshops will be used to share findings with organizations at the community level. Our targeted knowledge mobilization strategy will help ensure that our dissemination activities are accessible, relevant, and user-friendly to each of the different audiences.

## Discussion

### Expected Findings

This scoping review of reviews aims to map the current landscape, knowledge gaps, and areas for innovation to advance brain health and dementia research in Canada. More specifically, this review is anticipated to provide an overview of the current brain health and dementia research ecosystem. By consolidating existing research, this review will reveal gaps in knowledge and illuminate areas for future research. Although our review may highlight policy and practice implications, our study will be conducted to map the current research rather than assess research projects, policies, or practices. By revealing research gaps, our review’s findings will offer important insights for policymakers and research funders to help shed light on strategic areas for future research funding. Consequently, the findings from our study will fuel future investigations and illuminate priority research areas that may require implementation, monitoring, evaluation, and scaling up.

Our protocol adheres to the framework by Arksey and O’Malley [14] and the PRISMA-ScR guidelines [13], ensuring a rigorous, transparent, and systematic methodology. Guided by the criteria of trustworthiness by Lincoln and Guba [17], steps will be taken to ensure dependability, credibility, and transparency throughout the study. Although our research is not without limitations, the findings from our scoping review will be invaluable in helping identify critical research gaps and areas for innovation in the areas of dementia prevention, treatments, and quality of life. The findings from this study will provide essential information for prioritizing strategic funding initiatives and future directions to accelerate brain health and dementia research in Canada.

### Limitations

Although this scoping review of reviews will be conducted in a robust manner, it is important to note its limitations. For instance, as we will conduct a review of reviews, it is possible that some relevant studies will be excluded if they are not included in a review. Our selected electronic databases may also present limitations in that some relevant reviews may be overlooked if they are not indexed in the databases. Additionally, our search terms will not include each of the different types of dementia, which may limit the results of our search. Moreover, our search will only include reviews with Canadian authors, which may limit the generalizability of our findings. Accordingly, a future scoping review of reviews to examine the research on brain health and dementia worldwide would be informative. Another limitation is that our review only focuses on peer-reviewed publications from the years 2020 to 2025, which will only provide a snapshot in time. Consequently, future reviews could be augmented by ongoing updates to map the brain health and dementia research landscape longitudinally over time. Future researchers may consider developing an evolving scoping review of reviews to provide periodic updates to ensure that the evidence base remains timely and relevant for informing strategic priorities.

### Conclusions

Our scoping review of reviews will provide a robust synthesis of the brain health and dementia research landscape, helping document critical knowledge gaps and identify areas for innovation to advance the field. The results of this research will provide critical data to help inform strategic funding initiatives and future research directions. The findings from our scoping review will have implications for research funders, policymakers, community organizations, and researchers that are working to accelerate brain health and dementia research in Canada.

## Supplementary material

10.2196/79020 Checklist 1 PRISMA-ScR checklist.

## Abbreviations

PCC: population, concept, and context
PRISMA: Preferred Reporting Items for Systematic Reviews and Meta-Analyses
PRISMA-ScR: Preferred Reporting Items for Systematic Reviews and Meta-Analyses extension for Scoping Reviews
